# Differential effects of antiemetic serotonin receptor antagonist Ondansetron on nausea associated with *CHRM3* rs2165870 and *TACR1* rs3755468 single-nucleotide polymorphisms

**DOI:** 10.1186/s13041-025-01237-3

**Published:** 2025-07-21

**Authors:** Yuna Kang, Seii Ohka, Daisuke Nishizawa, Junko Hasegawa, Kyoko Nakayama, Kaori Yoshida, Kyotaro Koshika, Tatsuya Ichinohe, Kazutaka Ikeda

**Affiliations:** 1 Addictive Substance Project, Tokyo Metropolitan Institute of Medical Science, Setagaya-ku, Tokyo, 156-8506 Japan; 2 Department of Dental Anesthesiology, Tokyo Dental College, Chiyoda-ku, Tokyo, 101-0061 Japan; 3 Department of Neuropsychopharmacology, National Institute of Mental Health, National Center of Neurology and Psychiatry, Tokyo, 187-8553 Japan

**Keywords:** PONV, Nausea, Orthognathic surgery, Ondansetron, SNP

## Abstract

Postoperative nausea and vomiting (PONV) after orthognathic surgery is a serious postoperative complication. The cholinergic receptor muscarinic 3 (*CHRM3*) rs2165870 and tachykinin receptor 1 (*TACR1*) rs3755468 single-nucleotide polymorphisms (SNPs) have been reported to be involved in PONV. We evaluated the impact of these SNPs on PONV in a Japanese population who underwent orthognathic surgery under PONV prophylaxis with the 5-hydroxytryptamine (serotonin) receptor 3A receptor antagonist ondansetron. In 121 patients, dexamethasone was administered after intubation, followed by ondansetron before the end of surgery. An 11-point numeric rating scale (NRS) score for PONV (0–2 h or 2–24 h after anesthesia endpoint [a.a.e.]) and the presence or absence of metoclopramide administration (0–2 h or 2–24 h a.a.e.) were evaluated. If patients complained of PONV and had an NRS score ≥ 4, then metoclopramide was administered intravenously for PONV rescue. Patients were genotyped for the *CHRM3* rs2165870 and *TACR1* rs3755468 SNPs, followed by the statistical analysis of associations between these SNPs and phenotypes. AA carriers of *CHRM3* rs2165870 received metoclopramide at a significantly higher rate (*P* = 2.48 × 10^− 2^) and had higher NRS scores (*P* = 3.40 × 10^− 2^) under a diminished influence of ondansetron than GG and GA carriers. CC carriers of *TACR1* rs3755468 had significantly higher NRS scores under the sufficient influence of ondansetron than CT and TT carriers (*P* = 9.97 × 10^− 3^). Numeric rating scale scores showed a significant interaction between “time” (the effect of ondansetron) and “genotype” (two-way analysis of variance, *P* = 4.39 × 10^− 2^). AA carriers of *CHRM3* rs2165870 were significantly associated with “time” (*P* = 3.26 × 10^− 2^), and CC carriers of *TACR1* rs3755468 were not (*P* > 0.05). These results suggest that ondansetron significantly affects nausea that is associated with *CHRM3*, whereas it has a minimal effect on nausea that is associated with *TACR1*. This indicates that nausea that is associated with *CHRM3* is qualitatively different from nausea that is associated with *TACR1*. Ondansetron mainly exerts its effects outside the blood-brain barrier, which may lead to differential impacts on nausea that is associated with *CHRM3* and *TACR1*. These findings may provide future directions for tailor-made preventive measures against PONV that depend on high-risk genotypes of the *CHRM3* rs2165870 and *TACR1* rs3755468 SNPs.

**Supplementary Information:**

The online version contains supplementary material available at 10.1186/s13041-025-01237-3.

## Introduction

Postoperative nausea and vomiting (PONV) is one of the most common complications after major surgery. Orthognathic surgery has an even higher risk of PONV, the probability of which is 43–72% [1], because Le Fort I osteotomy, which involves detachment of the maxilla, causes a large amount of bleeding and makes it easy to swallow blood, which increases the risk of nausea. In orthognathic surgery, if vomiting occurs due to intermaxillary fixation postoperatively, then the risk of airway obstruction or aspiration pneumonia increases. Thus, the treatment of PONV is even more important. Risk factors for PONV have been reported to include female sex, a history of PONV and/or motion sickness, nonsmoking status, and young age [2]. Antiemetics for different receptors are recommended for patients with more than one risk factor for PONV [2]. Maintaining anesthesia with propofol is less likely to cause PONV than with volatile anesthetics [2]. In a previous study, the incidence of PONV was 15% when intraoperative anesthesia with propofol for orthognathic surgery was maintained [3]. In particular, there is strong evidence of efficacy of the combined administration of ondansetron (a selective [serotonin, 5-HT] receptor 3A [*5-HT3A receptor, HTR3A*] antagonist) and dexamethasone for PONV prophylaxis [2]. In our previous study, a combination of dexamethasone and ondansetron with a volatile anesthetic for orthognathic surgery resulted in a 9% occurrence of nausea [4]. Thus, even maintaining anesthesia with propofol or under the combined prophylactic administration of dexamethasone and ondansetron, some patients still experience nausea. Accordingly, we speculated that the risk of PONV involves genetic factors.

Nausea is a complex perceptual process that has different dimensions (e.g., learning, emotional, executive, motivational, or motor events), leading to a poor understanding of its mechanisms [5]. A systematic review identified four neural networks that are important for nausea [6]. First, the nucleus tractus solitarius (NTS) in the central nervous system (CNS) plays a pivotal role in nausea induction and receives inputs from the vestibular system, chemo- and mechano-receptive vagal afferents, and systemic agents. Second, serotonin activates chemical and/or mechanoreceptors that are expressed in the upper gastrointestinal tract, pharynx, and mediastinum, and these stimuli lead to the NTS via vagus nerve afferents. Third, systemic agents in the bloodstream, such as drugs, hormones, and neurotransmitters, reach the area postrema (chemoreceptor trigger zone [CTZ]) in the medulla oblongata outside the blood-brain barrier (BBB) and send signals to the NTS. Fourth, the NTS connects cerebral cortical areas, including the anterior cingulate cortex (ACC), through the basal ganglia in subcortical structures, leading to nausea. However, pathways that contribute to nausea in the CNS other than the brainstem and spinal cord have not been elucidated. According to the reported mechanism of vascular headache-related nausea and emesis, the trigeminovascular system connects centers that are associated with the generation of vascular headaches, nausea, emesis, autonomic activation, etc [7].

Cholinergic receptor muscarinic 3 (CHRM3), dopamine receptor D2 (DRD2), HTR3A, the µ-opioid receptor (OPRM1), and the neurokinin 1 receptor (tachykinin receptor 1 [TACR1]) are expressed in the CNS, area postrema, and vagus nerves and are reportedly involved in emesis via the area postrema and vagus nerves. Ondansetron predominantly suppresses emesis by antagonizing serotonin at the 5-HT_3A_ receptor on the afferent vagus nerve in the area postrema and gastrointestinal tract outside the BBB [6, 8], based on the fact that ondansetron cannot efficiently penetrate the BBB [9]. The pathway for emesis via the area postrema and vagus nerve that express CHRM3, DRD2, HTR3A, OPRM1, and TACR1 at least partially overlaps with the transduction pathway of ondansetron [8, 10, 11].

Among the genes of the above proteins that are related to emesis (CHRM3, DRD2, HTR3A, OPRM1, and TACR1), the *DRD2* rs1800497, *HTR3A* rs1176713, and *OPRM1* rs1799971 single-nucleotide polymorphisms (SNPs) were not independent risk factors for PONV [12], but some studies reported associations between the *CHRM3* rs2165870 and *TACR1* rs3755468 SNPs and PONV [13–16]. A genome-wide association study (GWAS) suggested that *CHRM3* rs2165870 contributed to a higher risk for PONV in the dominant model (GG + GA/AA) [13], and the AA genotype of *CHRM3* rs2165870 contributed to a higher risk for PONV in German or Chinese populations who underwent elective surgery [14–16]. Notably, the AA genotype of *CHRM3* rs2165870 was associated with a higher response to ondansetron in the Chinese Han population [15]. For *TACR1*, Hayase et al. suggested that the CC genotype of *TACR1* rs3755468 contributed to a higher risk for PONV in a Japanese population who underwent lower abdominal surgery without ondansetron administration [17]. To date, no studies have investigated effects of the *CHRM3* rs2165870 and *TACR1* rs3755468 SNPs on PONV in orthognathic surgery under PONV prophylaxis with ondansetron. In the present study, we evaluated the impact of the *CHRM3* rs2165870 and *TACR1* rs3755468 SNPs on PONV in a Japanese population who underwent orthognathic surgery under PONV prophylaxis with ondansetron and investigated effects of ondansetron on nausea that is associated with these SNPs.

## Materials and methods

### Patients who underwent orthognathic surgery under general anesthesia

**Fig. 1:**
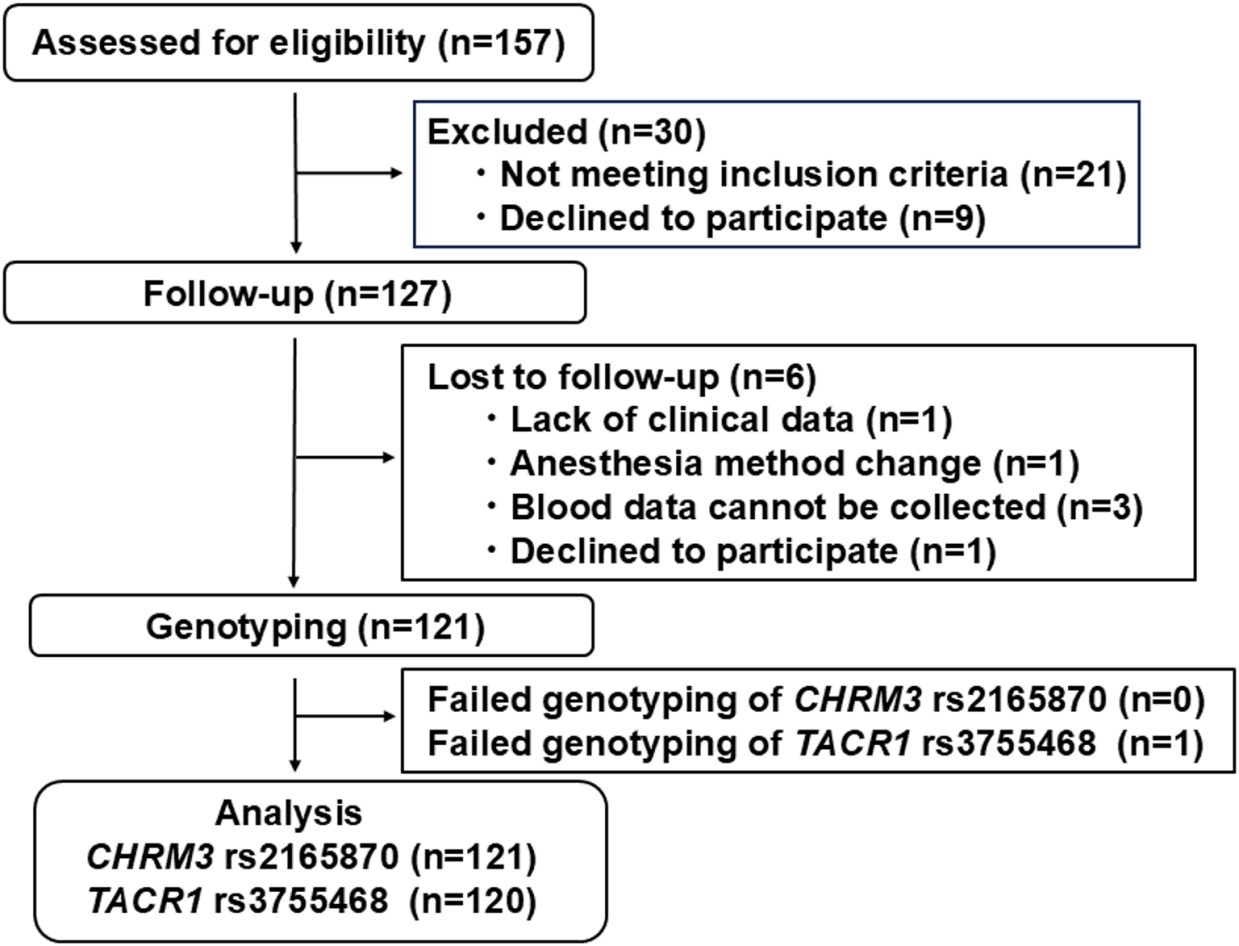
Flowchart for subject inclusion and exclusion in the study. Illustration of the process of enrolling patients for study inclusion

A total of 157 patients were recruited for participation in the study. However, 30 patients failed to meet the inclusion criteria, and another six patients were lost to follow-up after surgery, resulting in their exclusion (Fig. 1).

Consequently, enrolled in the study were 121 adult patients (American Society of Anesthesiologists Physical Status [ASA-PS] I-II, 18–50 years old, 48 males and 73 females) who were scheduled to undergo bimaxillary osteotomy (Le Fort I osteotomy) for jaw deformities under general anesthesia at The Tokyo Dental College Suidoubashi Hospital. Exclusion criteria included patients who underwent emergency surgery or had contraindications to ondansetron. All of the individuals who were included in the study were of Japanese descent. Peripheral blood samples were collected from these subjects for gene analysis. Detailed demographic and clinical data of the subjects are provided in Table 1 and Tables S1 and S2 in Additional file 1.

**Table 1:** Demographic and clinical data of patient subjects

	*n*	Minimum	Maximum	Mean	SD	Median
Sex						
Male	48					
Female	73					
Age (years)	121	18	50	26.64	8.15	27
Height (cm)	121	150	188	165.65	8.12	166.00
Weight (kg)	121	40	107	60.39	12.05	58.00
Body mass index (kg/m^2^)	121	16.02	35.75	21.89	3.40	21.05
ASA-PS						
I	103					
II	18					
Smoking history						
Absent	101					
Present	20					
Motion sickness						
Absent	78					
Present	43					
History of PONV						
Absent	110					
Present	11					
Apfel score^a^						
1	10					
2	34					
3	46					
4	31					
Operation time (min)	121	141	625	124.43	62.38	239.00
Anesthesia time (min)	121	202	690	305.6	64.26	299.00
Total infusion volume (ml)	121	1000	3350	1692.5	372.03	1600.00
Blood loss during surgery (ml)	121	36	835	210.25	134.28	186.00
Total remifentanil dose (µg/kg)^b^	121	42.02	153.03	76.98	21.95	74.63
Total intraoperative fentanyl dose (µg/kg)^b^	121	1.94	5.80	3.91	0.53	4.00
Postoperative fentanyl dose 2 h later (µg/kg)^b^	121	0	5.23	1.36	1.10	1.07
Postoperative fentanyl dose 24 h later (µg/kg)^b^	121	0	14.08	4.02	3.42	2.99
Nausea NRS (0–2 h)	121	0	10	0.56	1.46	0
Nausea NRS (2–24 h)	121	0	10	1.36	2.46	0
Metoclopramide administration (0–2 h)						
Absent	120					
Present	1					
Metoclopramide administration (2–24 h)						
Absent	93					
Present	28					
Vomiting (0–2 h)						
Absent	120					
Present	1					
Vomiting (2–24 h)						
Absent	115					
Present	6					

^a^Score for risk factors consisted of female sex, nonsmoking status, history of PONV or motion sickness, and postoperative opioid use

^b^Doses of opioids were normalized to body weight

The present study was approved by the Ethics Committees of Tokyo Dental College and Tokyo Metropolitan Institute of Medical Science (approval no. 1146 and 23 − 14 [1], respectively) and registered with the University Hospital Medical Information Network (registration no. UMIN000051727). The study was performed in accordance with provisions of the Declaration of Helsinki. All subjects provided written informed consent for the genetic studies.

### General anesthesia and nausea assessment

All participants received intubated general anesthesia according to the following protocol. After arrival in the operating room and starting standard anesthetic monitoring (noninvasive blood pressure, percutaneous oxygen saturation, electrocardiography, and capnography), peripheral intravenous (IV) access was established, and a continuous infusion of remifentanil (0.5 mg/kg/min) was initiated. The target-controlled infusion of propofol was started at 3.0 µg/ml according to the Japanese package insert. Infusion rates were maintained at 2.5–5.0 µg/ml, considering age and estimated brain concentration at loss of consciousness. After neuromuscular paralysis was achieved with rocuronium (0.6 mg/kg), nasotracheal intubation was performed. Following successful intubation, an IV bolus of dexamethasone (6.6 mg) was administered. An A-line was taken from the radial artery for invasive blood pressure measurement. Next, 10 ml of peripheral blood samples was collected from the radial artery for gene analysis. General anesthesia was maintained with propofol, oxygen (1 L/min), and air (3 L/min) along with a remifentanil infusion. Remifentanil infusion rates were started at 0.2 mg/kg/min and adjusted by 0.05 mg/kg/min every 5 min depending on changes in systolic blood pressure (20%) compared with values 5 min previously. At the time of maxillary transection, an IV bolus of fentanyl (2 µg/kg) was administered. At approximately 20 and 15 min before the end of surgery, an IV bolus of fentanyl (2 µg/kg) and ondansetron (4 mg) was administered, respectively. Following the completion of surgery, the degree of neuromuscular paralysis was assessed using a train-of-four monitor, and sugammadex (2–4 mg/kg) was administered as needed. Patients were extubated while awake after sufficient spontaneous ventilation and responsiveness to verbal commands were confirmed. After emergence from anesthesia and tracheal extubation, IV patient-controlled analgesia (PCA; 1 mg fentanyl in normal saline in a total volume of 50 ml) commenced using a CADD-Legacy PCA pump (Smiths Medical Japan, Tokyo, Japan) to prevent pain. The PCA settings included a bolus dose of fentanyl (50 µg) on demand with a lockout time of 10 min. Continuous background infusion was not used. Patient-controlled analgesia was continued for 24 h postoperatively. The use of intraoperative remifentanil, fentanyl, and postoperative PCA fentanyl during the 24 h postoperative period was recorded. The doses of remifentanil and fentanyl that were administered intraoperatively and postoperatively were normalized to body weight. For postoperative analgesia, acetaminophen and loxoprofen were administered on time in all patients.

Patient-rated nausea severity was assessed twice: 2 and 24 h after the anesthesia endpoint (a.a.e.) while avoiding bedtime using an 11-point numeric rating scale (NRS; 0 = no nausea, 10 = maximum imaginable nausea) score for PONV. Any episodes of vomiting were also recorded along with the timing. The NRS score was also evaluated when a patient complained of nausea or vomited. According to a previous study, dividing the PONV time into 0–2 h and 2–24 h a.a.e. allows determination of the efficacy of ondansetron [18]. Although the pharmacokinetic half-life of ondansetron in whole blood is ~ 6 h in Japanese individuals [19], it is preferable to investigate the effect of ondansetron during a period with a higher blood concentration of ondansetron (e.g., 0–2 h a.a.e.), similar to the previous study. Thus, the data-collecting split time point for PONV was set at 2 h a.a.e. in the present study. For patients without complaints of nausea or vomiting, NRS scores that were recorded 2 and 24 h a.a.e. were used for 0–2 h and 2–24 h a.a.e., respectively. For patients with complaints of nausea or vomiting, the maximum NRS scores that were recorded for each of the 0–2 h and 2–24 h a.a.e. were used. If the NRS score was ≥ 4 whenever a patient complained of nausea or vomited, IV metoclopramide (10 mg) was administered. If metoclopramide was administered, then the timing was recorded. Metoclopramide was administered up to twice daily.

Additional data that were gathered included patient demographics (sex, age, height, body weight, body mass index, ASA-PS, smoking history, motion sickness, and history of PONV) and surgical/anesthetic factors (operation time, anesthesia time, total infusion volume, blood loss, total remifentanil dose, total intraoperative fentanyl dose, total postoperative fentanyl dose, and timing of metoclopramide administration). We also calculated the number of PONV risk factors according to the Apfel score [20]: female sex, nonsmoker, history of PONV or motion sickness, and postoperative opioid use.

### Genotyping

Genomic DNA was extracted from whole-blood samples using standard procedures [15, 16]. The extracted DNA was dissolved in TE buffer (10 mM Tris-HCl and 1 mM ethylenediaminetetraacetic acid, pH 8.0). The DNA concentration was adjusted to 20 ng/µl for whole-genome genotyping using a NanoDrop ND-1000 Spectrophotometer (NanoDrop Technologies, Wilmington, DE, USA). The TaqMan allelic discrimination assay was conducted for genotyping the rs2165870 SNP of the *CHRM3* gene and rs3755468 SNP of the *TACR1* gene as described in previous reports [21, 22]. To perform the TaqMan assay with a LightCycler 480 II (Roche Diagnostics, Basel, Switzerland), we used TaqMan SNP Genotyping Assays (Life Technologies, Carlsbad, CA, USA) that contained sequence-specific forward and reverse primers to amplify the polymorphic sequence and two probes that were labeled with VIC and FAM dye to detect both alleles of the rs2165870 SNP of the *CHRM3* gene and rs3755468 SNP of the *TACR1* gene (Assay ID: C_9774837_30 and C_27474834_20, respectively). Real-time polymerase chain reaction was performed in a final volume of 10 µl that contained 2× LightCycler 480 ІІ Probes Master (Roche Diagnostics), 40× TaqMan SNP Genotyping Assays, 5–50 ng genomic DNA as the template, and H_2_O (Roche Diagnostics). The thermal conditions were the following: 95 °C for 10 min, followed by 45 cycles of 95 °C for 10 s and 60 °C for 60 s, with final cooling at 50 °C for 30 s. Afterward, endpoint fluorescence was measured for each sample well, and each genotype was determined based on the presence or absence of each type of fluorescence. We performed TaqMan SNP Genotyping Assays on 121 samples, although we failed to genotype one sample for *TACR1* rs3755468 (Fig. 1). Raw genotype data for clinical phenotypes on the *CHRM3* rs2165870 and *TACR1* rs3755468 SNPs are shown in Dataset S1.

### Power analysis

In a previous study, the patient numbers of each genotype of *CHRM3* rs2165870 with PONV at 2–6 h a.a.e. were GG = 82, GA = 96, and AA = 31. Without PONV at 2–6 h a.a.e., they were GG = 137, GA = 148, and AA = 18 [23]. Based on previous studies, an a priori power analysis was performed with G*Power (version 3.1.9.6, Heinrich Hein University), and an effect size of 0.26 in Cohen’s conventional small effect size was used for calculation [24, 25]. Therefore, we set a significance level of 5% and power of 80% and calculated a sample size of 117 total patients.

### Statistical analysis

The samples (121 and 120) were analyzed for the *CHRM3* rs2165870 SNP and *TACR1* rs3755468 SNP, respectively (Fig. 1). For all genotype frequency data, deviations from the theoretical Hardy-Weinberg equilibrium (HWE) distribution were examined, and *χ*^*2*^ tests, one-way analysis of variance (ANOVA), *t*-test, two-way ANOVA, and Sidak’s multiple-comparison *post hoc* test were performed to analyze associations with the clinical data. The HWE distribution test was performed using Haploview 4.2 and Calculator of Hardy-Weinberg equilibrium [26]. Hardy-Weinberg equilibrium was tested using the *χ*^*2*^ test (*df* = 1) for genotypic distributions of the *CHRM3* rs2165870 and *TACR1* rs3755468 SNPs with values of significant deviation set to *P* = 0.05. The *χ*^*2*^ test, one-way ANOVA, and *t*-test were performed using SPSS 28 software (IBM Japan, Tokyo, Japan). Two-way ANOVA and Sidak’s multiple-comparison *post hoc* test were performed using GraphPad Prism 7.00 software. Bonferroni correction for multiple comparisons was performed for two SNPs of *CHRM3* rs2165870 and *TACR1* rs3755468 in the genotypic, dominant, and recessive models. Mean NRS scores are expressed as the mean ± SD and were analyzed using one-way ANOVA or *t*-test. Bonferroni correction for multiple comparisons was performed for two SNPs of *CHRM3* rs2165870 and *TACR1* rs3755468 in the genotypic, dominant, and recessive models. *P* < 0.05 was considered a significant association, and 0.05 ≤ *P* < 0.10 was considered a trend toward an association.

### Public database search

We extracted information on expression quantitative trait loci (eQTLs) of the SNPs using the GTEx Portal to examine effects of the SNP on gene expression levels in humans [27]. We also extracted information on expression splicing quantitative trait loci (sQTLs) of the SNPs using the GTEx Portal to examine effects of the SNPs on gene expression levels in humans [28]. We extracted information on CHRM3 and TACR1 mRNA expression in human tissues and cells using The Human Protein Atlas [29]. We extracted the DNase-Seq signal of the genes’ genomic regions and transcript of the genes’ genomic regions and antisense noncoding RNA using the ZENBU genome browser on December 9, 2024, to investigate transcriptional regulation around SNP regions in the genes [30, 31]. The data source of the DNase-Seq signal was the NIH Roadmap Epigenomics Mapping Consortiums in 127 samples, showing only a *P*-value signal ≥ 2. We searched the transcripts starting from around the SNP using the National Center for Biotechnology Information (NCBI) [32]. We investigated allele frequencies by regional population using the NCBI database [33, 34]. The nucleotide sequence of the human genome assembly hg19 GRCh37 from Genome Reference Consortium was used.

### Results

### Background characteristics of the patients related to surgery and anesthesia

In the present study, we analyzed 121 and 120 patients for the *CHRM3* rs2165870 and *TACR1* rs3755468 SNPs, respectively, who underwent general anesthesia for orthognathic surgery. The background characteristics of the patients related to the surgery and anesthesia are shown in Table 1 and Tables S1 and S2 in Additional file 1.

### *CHRM3* rs2165870 and *TACR1* rs3755468 SNPs were in HWE

The genotypic distribution of *CHRM3* rs2165870 and *TACR1* rs3755468 did not significantly deviate from the theoretical HWE in the patient group (*CHRM3* rs2165870: *P* = 0.59, *χ*^*2*^ = 0.2925; *TACR1* rs3755468: *P* = 0.93, *χ*^*2*^ = 0.0083).

### *CHRM3* rs2165870 SNP was associated with Metoclopramide administration 0–24 h and 2–24 h a.a.e.

Based on the previous report that *CHRM3* rs2165870 is associated with PONV in the Chinese Han population [23], we investigated whether the *CHRM3* rs2165870 is associated with PONV in the present study in a Japanese population. In 0–24 h a.a.e., the *CHRM3* rs2165870 SNP showed a significant association with metoclopramide administration in the recessive model (*χ*^*2*^ test, *P* = 3.01 × 10^− 2^; Table 2), suggesting that *CHRM3* rs2165870 is associated with metoclopramide administration in the Japanese population, consistent with the previous results in the Chinese Han population.

**Table 2:** Association between genotypes of *CHRM3* rs2165870 SNP and Metoclopramide administration (recessive model, *χ*^*2*^ test)

Hours after anesthesia endpoint	Metoclopramide administration	Genotypes		*P*
		GG *+* GA (*n* = 117*)*	AA (*n* = 4)	
0–2	with	1 (1%)	0 (0%)	1.00 × 10^− 1^
	without	116 (99%)	4 (100%)	
2–24	with	25 (21%)	3 (75%)	2.48 × 10^− 2*^
	without	92 (79%)	1 (25%)	
0–24	with	26 (22%)	3 (75%)	3.01 × 10^− 2*^
	without	91 (78%)	1 (25%)	

^*^P < 0.05

Because *CHRM3* rs2165870 showed no significant association in the genotypic or dominant models in 0–24 h a.a.e. (*χ*^*2*^ test, *P* > 0.05; Tables S3 and S4 in Additional file 1), we thereafter performed an analysis mainly in the recessive model for *CHRM3* rs2165870.

Next, because *CHRM3* rs2165870 is reportedly associated with the efficacy of ondansetron in the Chinese Han population [18], we investigated whether *CHRM3* rs2165870 is associated with effects of ondansetron in the present study in the Japanese population. According to the previous study, dividing the time of PONV into 0–2 h and 2–24 h a.a.e. to determine the efficacy of ondansetron [18], we divided the patients into 0–2 h and 2–24 h a.a.e. with the timing of metoclopramide administration (NRS score ≥ 4). The influence of ondansetron is supposed to be sufficient in 0–2 h a.a.e. and diminished in 2–24 h a.a.e. To examine the effect of ondansetron, we analyzed the association between *CHRM3* rs2165870 and metoclopramide administration in 0–2 h and 2–24 h a.a.e. In 0–2 h a.a.e., the *CHRM3* rs2165870 SNP showed no association with metoclopramide administration (*χ*^*2*^ test, *P* > 0.05; Table 2), whereas *CHRM3* rs2165870 showed a significant association with metoclopramide administration in 2–24 h a.a.e. (*χ*^*2*^ test, *P* = 2.48 × 10^− 2^; Table 2). The pharmacokinetic half-life of ondansetron in whole blood is approximately 6 h in Japanese individuals [19]; thus, the significant association that was observed under the diminished influence of ondansetron (2–24 h a.a.e.) and not under a greater effect of ondansetron (0–2 h a.a.e.) suggests that *CHRM3* rs2165870 is associated with the effect of ondansetron.

The previous study suggested that the AA genotype of *CHRM3* rs2165870 was associated with a higher risk of PONV and showed a response to ondansetron treatment [18]. To investigate whether the AA genotype of *CHRM3* rs2165870 is associated with a higher risk of PONV and shows a response to ondansetron treatment in the present study, we analyzed the genotype distribution in the recessive model (Table 2). In 2–24 h a.a.e., the AA genotype had a higher rate of metoclopramide administration than GG + GA genotypes (2–24 h a.a.e.; GG + GA/AA with metoclopramide, 21%/75%, without metoclopramide, 79%/25%; Table 2). The results suggest that the AA genotype of *CHRM3* rs2165870 has a higher risk of PONV and response to ondansetron treatment, consistent with the results of the previous study.

Altogether, under the sufficient influence of ondansetron (0–2 h a.a.e.), no significant association was observed between *CHRM3* rs2165870 and metoclopramide administration, whereas under the diminished influence of ondansetron (2–24 h a.a.e.), there was a significant need for metoclopramide administration in patients with the AA genotype of *CHRM3* rs2165870. These results suggest that patients with the AA genotype of *CHRM3* rs2165870 are more prone to experience nausea under the diminished influence of ondansetron.

### *CHRM3* rs2165870 SNP was significantly associated with NRS score 2–24 h a.a.e.

To further confirm consistency with the previous results of the association between *CHRM3* rs2165870 and PONV or the efficacy of ondansetron, we investigated whether there was a significant association between *CHRM3* rs2165870 and the patients’ mean NRS scores in 0–2 h, 2–24 h, or 0–24 h a.a.e. In 0–24 h a.a.e., *CHRM3* rs2165870 showed a trend toward an association in the recessive model (*t*-test, *P* = 7.95 × 10^− 2^; Table 3).

**Table 3:** Association between genotypes of *CHRM3* rs2165870 SNP and NRS scores (recessive model, *t*-test)

Hours after anesthesia endpoint	NRS values (mean ± SD)		*P*
	GG + GA	AA	
0-2^a^	0.57 ± 1.27	0.00 ± 0.00	8.79 × 10^− 1^
2-24^a^	1.38 ± 2.45	4.25 ± 2.59	3.40 × 10^− 2*^
0-24^b^	1.61 ± 2.50	4.25 ± 2.59	7.95 × 10^− 2♰^

^*^P < 0.05

^†^0.05 < *P* < 0.10

^a^NRS score assessed during indicated hours after anesthesia

^b^Maximum NRS score in 0–24 h

To examine the effect of ondansetron, we analyzed the association between *CHRM3* rs2165870 and mean NRS scores in 0–2 h a.a.e. (sufficient influence of ondansetron) and 2–24 h a.a.e. (less sufficient influence of ondansetron). In 0–2 h a.a.e., *CHRM3* rs2165870 showed no association with mean NRS scores (*t*-test, *P* > 0.05; Table 3), whereas in 2–24 h a.a.e., *CHRM3* rs2165870 showed a significant association with mean NRS scores in the recessive model (*t*-test, *P* = 3.40 × 10^− 2^; Table 3). No significant association was found in 0–2 h, 2–24 h, or 0–24 h a.a.e. in the genotypic or dominant model (one-way ANOVA or *t*-test, *P* > 0.05; Tables S5 and S6 in Additional file 1). The results suggest that *CHRM3* rs2165870 does not show a significant association with mean NRS scores under the sufficient influence of ondansetron (0–2 h a.a.e.), whereas this SNP showed a significant association with mean NRS scores under the diminished influence of ondansetron (2–24 h a.a.e.), confirming that *CHRM3* rs2165870 is associated with PONV and the efficacy of ondansetron.

### *TACR1* rs3755468 SNP showed no significant association with Metoclopramide administration

Based on the previous report that *TACR1* rs3755468 is associated with PONV in the Japanese population [17], we investigated whether *TACR1* rs3755468 is associated with PONV in the present study in the Japanese population. *TACR1* rs3755468 showed no significant association with metoclopramide administration in 0–2 h, 2–24 h, or 0–24 h a.a.e. (genotypic, dominant, and recessive models: *P* > 0.05; Table 4, Tables S7 and S8 in Additional file 1), although *TACR1* rs3755468 showed a trend toward an association with metoclopramide administration in 0–24 h a.a.e. in the dominant model (*χ*^*2*^ test, *P* = 6.05 × 10^− 2^; Table 4).

**Table 4:** Association between genotypes of *TACR1* rs3755468 SNP and Metoclopramide administration (dominant model, *χ*^*2*^ test)

Hours after anesthesia endpoint	Metoclopramide administration	Genotypes		*P*
		CC (*n* = 36)	CT + TT (*n* = 84)	
0–2	with	1 (3%)	0 (0%)	2.50 × 10^− 1^
	without	35 (97%)	84 (100%)	
2–24	with	12 (33%)	15 (18%)	1.26 × 10^− 1^
	without	24 (67%)	69 (82%)	
0–24	with	13 (36%)	15 (18%)	6.05 × 10^− 2†^
	without	23 (64%)	69 (82%)	

^†^0.05 < *P* < 0.10

The results suggested that *TACR1* rs3755468 showed a trend toward an association with PONV in 0–24 h a.a.e. in the present study, partially consistent with the previous report. Because *TACR1* rs3755468 showed a trend toward an association with metoclopramide administration in 0–24 h a.a.e. in the dominant model, we mainly focused thereafter on the dominant model for *TACR1* rs3755468.

### *TACR1* rs3755468 SNP was significantly associated with NRS score in 0–2 h a.a.e.

To further examine the association between *TACR1* rs3755468 and PONV or the efficacy of ondansetron, we investigated whether there was a significant association between *TACR1* rs3755468 and patients’ NRS scores in 0–2 h, 2–24 h, or 0–24 h a.a.e. In 0–24 h a.a.e., *TACR1* rs3755468 showed a trend toward an association with the mean maximum NRS score in the dominant model (*t*-test, *P* = 8.07 × 10^− 2^; Table 5).

**Table 5:** Association between genotypes of *TACR1* rs3755468 SNP and NRS scores (dominant model, *t*-test)

Hours after anesthesia endpoint		NRS values (mean ± SD)			*P*
		CC	CT + TT		
0-2^a^		1.14 ± 2.16	0.32 ± 0.94		9.97 × 10^− 3^*
2-24^a^		1.81 ± 2.55	1.08 ± 2.41		3.40 × 10^− 1^
0-24^b^		2.33 ± 2.87	1.05 ± 2.09		8.07 × 10^− 2♰^

^*^P < 0.05

^†^0.05 < *P* < 0.10

^a^NRS score assessed during indicated hours after anesthesia

^b^Maximum NRS score in 0–24 h

To examine the effect of ondansetron, we analyzed the association in 0–2 h and 2–24 h a.a.e. In 0–2 h a.a.e., *TACR1* rs3755468 showed a significant association with the mean NRS score in the dominant model (*t*-test, *P* = 9.97 × 10^− 3^; Table 5), whereas in 2–24 h a.a.e., *TACR1* rs3755468 did not show a significant association with the mean NRS score in the dominant model (*t*-test, *P* > 0.05; Table 5). In the genotypic and recessive models, *TACR1* rs3755468 showed no significant association (one-way ANOVA or *t*-test, *P* > 0.05; Tables S9 and S10 in Additional file 1), except for showing a significant association in the genotypic model in 0–2 h a.a.e. (one-way ANOVA, *P* = 3.74 × 10^− 2^; Table S9 in Additional file 1). These results suggest that the *TACR1* rs3755468 showed a significant association with PONV under the sufficient influence of ondansetron (0–2 h a.a.e.), indicating that *TACR1* rs3755468 is not associated with the effect of ondansetron.

The previous study suggested that the CC genotype of *TACR1* rs3755468 was associated with a higher risk of PONV [17]. To investigate whether the CC genotype of *TACR1* rs3755468 is associated with a higher risk of PONV in the present study, we analyzed the genotype distribution in the dominant model (Table 5). In 0–2 h a.a.e., CC carriers showed a higher mean NRS score than CT + TT carriers (CC, 1.14 ± 2.16; CT + TT, 0.32 ± 0.94; Table 5). These results suggest that CC carriers of *TACR1* rs3755468 had a significantly higher NRS score under the sufficient influence of ondansetron (0–2 h a.a.e.), suggesting that individuals with the CC genotype of *TACR1* rs3755468 are more prone to nausea without being associated with the effect of ondansetron.

### Nausea related to *CHRM3* is qualitatively different from nausea related to *TACR1*

**Fig. 2:**
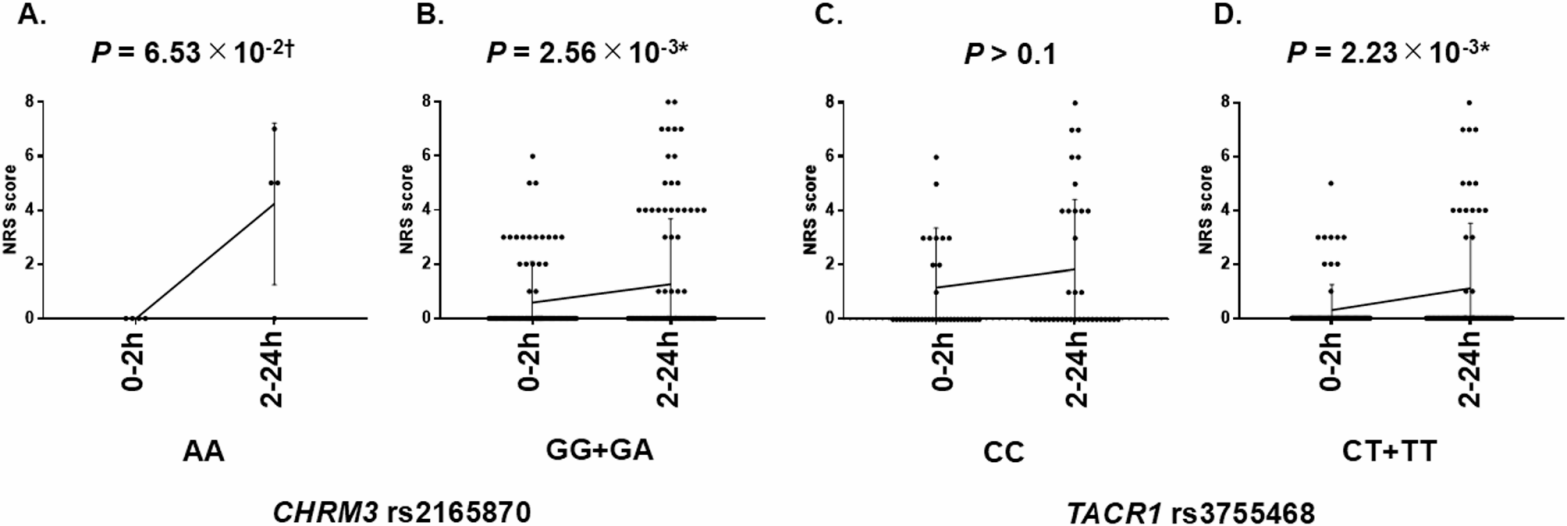
Associations between NRS scores 0–2 h and 2–24 h after anesthesia endpoint in genotypes of *CHRM3* rs2165870 and *TACR1* rs3755468 SNPs. We analyzed associations between NRS scores 0–2 h and 2–24 h after anesthesia endpoint (a.a.e.) in (**A**) AA and (**B**) AG + GG genotypes of *CHRM3* rs2165870 and (**C**) CC and (**D**) CT + TT genotypes of *TACR1* rs3755468 SNPs (*t*-test). The AA genotype of *CHRM3* rs2165870 showed a trend toward an association with an increasing mean NRS score over time (**A**), whereas the CC genotype of *TACR1* rs3755468 showed an unchanged mean NRS score over time (**C**), suggesting that the AA genotype of *CHRM3* rs2165870 has a tendency toward the suppression of nausea with ondansetron, whereas the CC genotype of *TACR1* rs3755468 does not respond to ondansetron sufficiently for nausea suppression. The 95% confidence intervals were (**A**) -9.00 to 0.50, (**B**) -1.12 to -0.25, (**C**) -1.60 to 0.27, and (**D**) -1.32 to -0.30. Each plot represents a particular patient. Error bars indicate the SD. ^*^P < 0.05, ^†^0.05 < *P* < 0.10

To analyze the effect of time course a.a.e. on nausea, we analyzed the association between 0-2 h or 2–24 h a.a.e. and mean NRS scores in genotypes of the *CHRM3* rs2165870 and *TACR1* rs3755468 SNPs. The AA genotype of *CHRM3* rs2165870 showed a trend toward an association with an increasing mean NRS score over time (mean NRS score: 0–2 h a.a.e., 0.00 ± 0.00; 2–24 h a.a.e., 4.25 ± 2.59; *t*-test, *P* = 6.53 × 10^− 2^; Table 3; Fig. 2), whereas the CC genotype of *TACR1* rs3755468 showed an unchanged mean NRS score over time (mean NRS score: 0–2 h a.a.e., 1.14 ± 2.16; 2–24 h a.a.e., 1.81 ± 2.55; *t*-test, *P* > 0.1; Table 5; Fig. 2). These results suggest that the AA genotype of *CHRM3* rs2165870 has a tendency toward suppressing nausea with ondansetron, whereas the CC genotype of *TACR1* rs3755468 does not respond to ondansetron sufficiently for nausea suppression. In GG + GA genotypes of *CHRM3* rs2165870 and CT + TT genotypes of *TACR1* rs3755468, the mean NRS scores significantly increased over time (*CHRM3* rs2165870 GG + GA, mean NRS score: 0–2 h a.a.e., 0.57 ± 1.27; 2–24 h a.a.e., 1.38 ± 2.45; *t*-test, *P* = 2.56 × 10^− 3^; *TACR1* rs3755468 CT + TT, mean NRS score: 0–2 h a.a.e., 0.32 ± 0.94; 2–24 h a.a.e., 1.08 ± 2.41; *t*-test, *P* = 2.23 × 10^− 3^; Tables 3 and 5; Fig. 2).

To further examine differences in nausea related to *CHRM3* rs2165870 and *TACR1* rs3755468, we analyzed the association among the AA genotype of *CHRM3* rs2165870, CC genotype of *TACR1* rs3755468, and mean NRS scores in 0–2 h and 2–24 h a.a.e. The two-way mixed-effect ANOVA showed the following: interaction, *P* = 4.39 × 10^− 2^, effect of “time”, *P* = 9.4 × 10^− 3^; effect of “genotype”, *P* > 0.05. These results clearly showed that mean NRS scores were significantly associated with time, with a significant “time” × “genotype” interaction. Sidak’s multiple-comparison *post hoc* test showed that the AA genotype of *CHRM3* rs2165870 was significantly associated with time (*P* = 3.26 × 10^− 2^), but the CC genotype of *TACR1* rs3755468 was not (*P* > 0.05). These results suggest that nausea that is related to *CHRM3* is qualitatively different from nausea that is related to *TACR1*.

### Public database results on *CHRM3* rs2165870

**Fig. 3:**
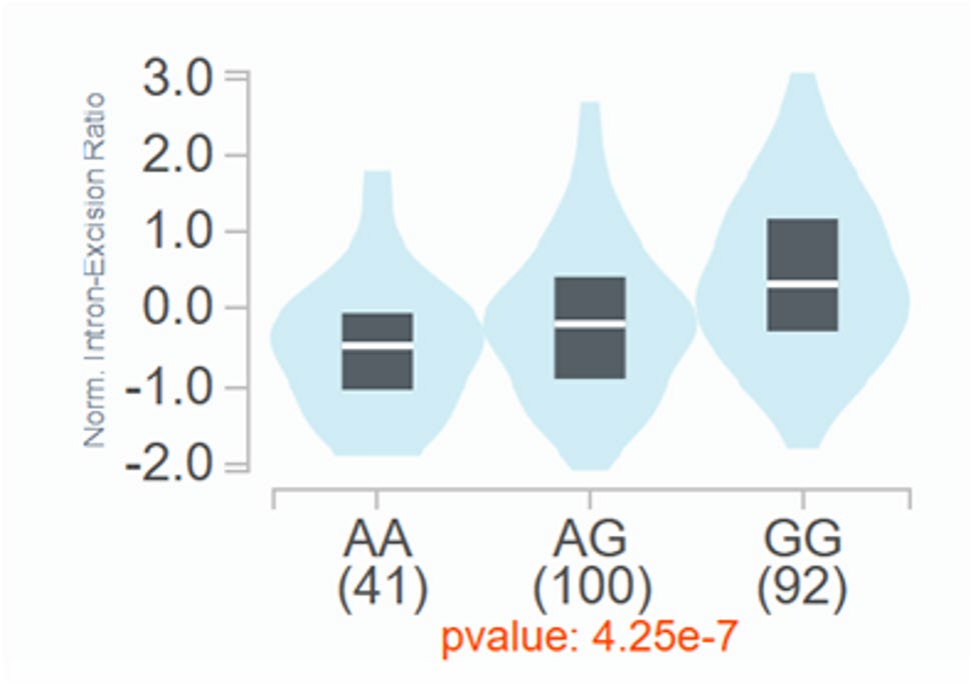
*CHRM3* expression levels for each genotype of *CHRM3* rs2165870 after splicing in the anterior cingulate cortex. *CHRM3* rs2165870 is located at a splicing quantitative trait locus (*sQTL*) at 239795586:239841486 on GRCh37 (239632286:239678186 on GRCh38) in the anterior cingulate cortex (ACC) [28]. Figure adapted from GTEx Portal (public domain).

**Fig. 4:**
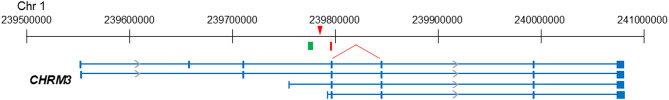
*CHRM3* rs2165870 affects splicing, and transcripts start from around *CHRM3* rs2165870. Representative transcripts of *CHRM3* are shown. A representative short transcript starts from around 10 kilobases (kb) downstream of *CHRM3* rs2165870. *CHRM3* rs2165870 (location on chromosome 1: 239785420) is located at the red triangle. The red rectangle represents the open chromatin region on the genome around *CHRM3* rs2165870 in blood vessels, stomach, and small intestine. The green rectangle represents the enhancer region on the genome around *CHRM3* rs2165870 in the small intestine and stomach. *CHRM3* rs2165870 is located at a splicing quantitative trait locus (sQTL) at 239795586:239841486, indicated in the red polyline between the exons above the gene. The numbers on the upper side of the figure are locations on GRCh37 on chromosome 1. Blue boxes represent the exon, and blue lines represent the intron

To investigate the function of *CHRM3* rs2165870, we searched public databases. According to the Adult Genotype-Tissue Expression (GTEx) project, *CHRM3* rs2165870 is located at a splicing quantitative trait locus (sQTL) at 239795586:239841486 on GRCh37 (239632286:239678186 on GRCh38) (Figs. 3 and 4) and 239795586:239797623 on GRCh37 (239632286:239634323 on GRCh38) in the human ACC [28]. Transcripts with splicing at 239795586:239841486 increase as the number of G alleles in the genotype increases (Fig. 3). For transcripts with splicing at 239795586:239797623, we could not confirm the transcript with splicing that has been reported to date. The results suggest that *CHRM3* rs2165870 manipulates *CHRM3* expression through an alteration of splicing in the ACC.

Furthermore, according to the FANTOM CAT database in the ZENBU genome browser, DNase-seq results in 10 kilobases (kb) upstream and downstream of *CHRM3* rs2165870 showed an open chromatin region around the SNP on the genome in blood vessels, stomach, and small intestine (*P*_value_signal: blood vessels, 2.23; stomach, 2.57; small intestine, 4.28; Fig. 4 and Table S11 in Additional file 2), and an enhancer region exists around the SNP on the genome in the small intestine and stomach (Fig. 4 and Table S12 in Additional file 3) [30]. According to the NCBI database, three short transcripts start from around 10 kb downstream of *CHRM3* rs2165870 (Fig. 4, transcript variants 2, X21, and X13), producing full-length CHRM3 protein [32]. These results suggest that the short mRNAs would be expressed from around *CHRM3* rs2165870 in these human tissues (blood vessels, stomach, and small intestine), followed by the expression of functional CHRM3 protein.

### Public database results on *TACR1* rs3755468

**Fig. 5:**
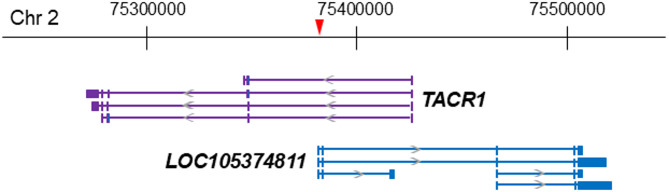
Transcripts of antisense *LOC105374811* start around *TACR1* rs3755468 and overlap with the transcription start site of *TACR1*. Representative transcripts of *TACR1* and *LOC105374811* are shown. *TACR1* rs3755468 (location on chromosome 2: 75382391) exists around 1 kb downstream of the transcription start site of *LOC105374811* (Table S13 in Additional file 4). The long transcripts of *LOC105374811* overlap with the transcription start site of *TACR1*. The numbers on the upper side of the figure are locations on GRCh37 on chromosome 2. Blue or purple boxes represent the exon, and blue or purple lines represent the intron

To investigate the function of the *TACR1* rs3755468, we searched public databases. According to the FANTOM CAT database in the ZENBU genome browser, some of the transcripts of the antisense noncoding RNA *LOC105374811* gene (Ensembl: ENSG00000270571) span the transcription start site of *TACR1* mRNA (location of the mRNA start: 75426645; Fig. 5 and Table S13 in Additional file 4) [31]. These *LOC105374811* transcripts are expressed from around 1 kb upstream of *TACR1* rs3755468 (location of the transcripts start: 75381413, 75381443, 75381490, and 75381512; Fig. 5 and Table S13 in Additional file 4).

**Fig. 6:**
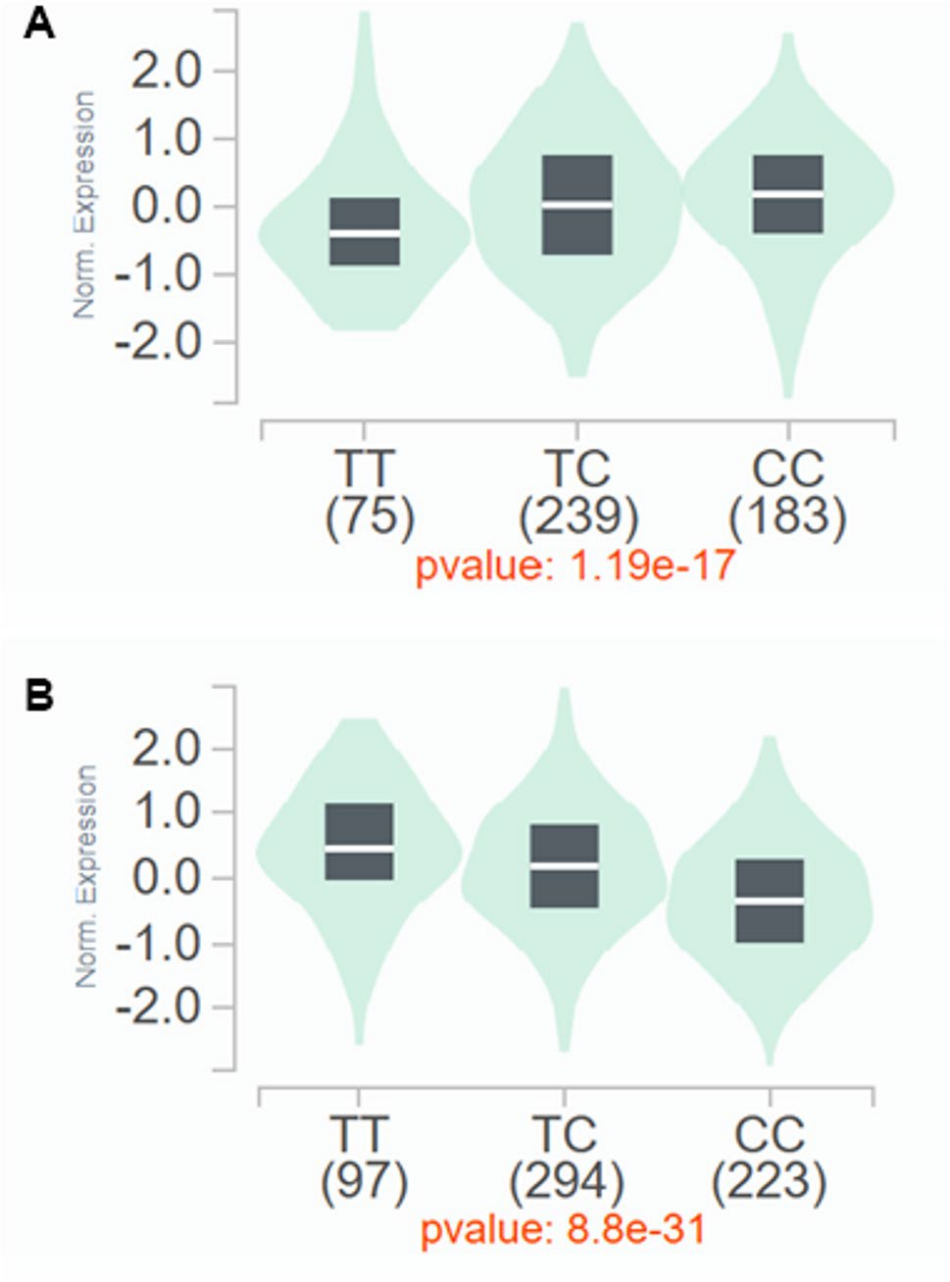
*TACR1* and antisense RNA *LOC105374811* expression levels in each genotype of *TACR1* rs3755468. *TACR1* rs3755468 is located at an expression quantitative trait locus (eQTL) for *TACR1* (**A**) and *LOC105374811* (**B**) [27]. Figure adapted from GTEx Portal (public domain)

According to the GTEx project, the *TACR1* rs3755468 is located at an expression quantitative trait locus (eQTL) for *TACR1* (Fig. 6A) and *LOC105374811* (Fig. 6B) [27]. As the number of C alleles in genotypes of *TACR1* rs3755468 increases, the expression of antisense RNA *LOC105374811* decreases, whereas *TACR1* mRNA increases (Fig. 6). The data suggest that more antisense RNA *LOC105374811* is transcribed when more *TACR1* transcription is inhibited. This raises the possibility that antisense RNA *LOC105374811* expression in CC carriers of *TACR1* rs3755468 is lower than in the other genotypes, leading to higher *TACR1* mRNA transcription.

## Discussion

Genetic factors are suggested to be risk factors for PONV [35]. We inferred that genetic factors are responsible for the appearance of PONV even after the combined prophylactic administration of dexamethasone, ondansetron, and propofol during general anesthesia. Among the genes that are involved in the mechanism of nausea through the area postrema and vagus nerve, which are related to the mechanism of action of ondansetron, we focused on the *CHRM3* and *TACR1* genes, which have SNPs that are significantly associated with nausea. In the present study, we evaluated the influence of the *CHRM3* rs2165870 and *TACR1* rs3755468 SNPs on PONV in a Japanese population who underwent orthognathic surgery under PONV prophylaxis with ondansetron and evaluated the different effects of ondansetron on nausea that is associated with these SNPs.

*AA genotype of CHRM3 rs2165870 is associated with a higher risk of nausea after orthognathic surgery*,* and ondansetron is effective for preventing nausea*.

*CHRM3* rs2165870 had no significant association with metoclopramide administration in 0–2 h a.a.e. under the sufficient influence of ondansetron, whereas patients with the AA genotype of *CHRM3* rs2165870 had a significant need for metoclopramide administration in 2–24 h a.a.e. under the insufficient influence of ondansetron. Moreover, *CHRM3* rs2165870 had no significant association with mean NRS scores in 0–2 h a.a.e. under the sufficient influence of ondansetron, whereas patients with the AA genotype of *CHRM3* rs2165870 had a significantly higher mean NRS score in 2–24 h a.a.e. under the insufficient influence of ondansetron (mean NRS score: 0–2 h a.a.e., 0.00; 2–24 h a.a.e., 4.25; Table 3). These results suggest that AA carriers of *CHRM3* rs2165870 have a higher risk of nausea after orthognathic surgery while ondansetron is effective. In AA carriers of *CHRM3* rs2165870, because nausea tended to occur under the insufficient influence of ondansetron, additional ondansetron administration is recommended.

A few studies have reported on *CHRM3* rs2165870 in Chinese populations. Wang et al. and Cai et al. reported that *CHRM3* rs2165870 contributed to a higher risk for PONV in AA carriers in Chinese populations [16, 23], consistent with the present findings that patients with the AA genotype of *CHRM3* rs2165870 has a higher risk of nausea after orthognathic surgery (Tables 2 and 3). Wang et al. also reported that AA carriers of *CHRM3* rs2165870 were associated with a higher response to ondansetron in the Chinese Han population [18], consistent with the present findings that ondansetron was effective for preventing nausea among individuals with the AA genotype of *CHRM3* rs2165870 (Tables 2 and 3).

According to the GTEx portal, *CHRM3* rs2165870 is located at an sQTL in the ACC (Fig. 3) [28], which is involved in nausea [6]. This implies that *CHRM3* rs2165870 could manipulate *CHRM3* expression through an alteration of splicing in the ACC. Furthermore, an open chromatin region, which contains regulatory regions, exists around the SNP on the genome in blood vessels, the small intestine, and the stomach. Together with the fact that the short transcripts start from around *CHRM3* rs2165870, producing full-length CHRM3 [32], the short mRNAs would be expressed downstream from *CHRM3* rs2165870 in these human tissues [29]. The amount of short mRNA expression might depend on the particular genotype of *CHRM3* rs2165870. For CHRM3 expression in human tissues, it is expressed at low to medium levels in the gastrointestinal tract according to The Human Protein Atlas [29]. Because stimulating blood vessels and the gastrointestinal tract leads to nausea [36], higher CHRM3 expression in blood vessels and/or the gastrointestinal tract could easily lead to nausea, depending on the particular genotype of *CHRM3* rs2165870.

Allele frequencies of *CHRM3* rs2165870 in the present study were the following: G-allele frequency = 80%, A-allele frequency = 20%. According to the NCBI database, the allele frequencies in the present study are similar to the East Asian population (G-allele frequency = 81%, A-allele frequency = 19%). Other regional populations have some variation (e.g., American population: G allele frequency = 67%, A allele frequency = 33%, European population: G allele frequency = 66%, A allele frequency = 34%, African population: G allele frequency = 98%, A allele frequency = 2%) [33]. Notably, the African population has less variation and is almost homogenous in allele frequency. The A-allele frequency in the present study was 20%, whereas in the African population, it is 2%. Thus, few AA carriers of *CHRM3* rs2165870 exist in the African population, making it difficult to assess the risk of PONV according to *CHRM3* rs2165870 in the African population. In the African population, because the proportion of AA carriers of *CHRM3* rs2165870 is lower than in other regional populations, the proportion that has a PONV risk that is associated with *CHRM3* rs2165870 is lower than in other regional populations according to the present results.

*CC genotype of TACR1 rs3755468 is associated with a higher risk of nausea after orthognathic surgery*,* responding less to ondansetron for preventing nausea*.

Hayase et al. reported that *TACR1* rs3755468 contributed to a higher risk of PONV in CC carriers in Japanese populations who underwent lower abdominal surgery [17], which is consistent with the present findings that CC carriers of *TACR1* rs3755468 had a higher risk of nausea after orthognathic surgery (Table 5).

CC carriers of *TACR1* rs3755468 had a significantly higher mean NRS score than carriers of other genotypes in both 0–2 and 2–24 h a.a.e., regardless of the influence of ondansetron (Table 5). The results suggest that CC carriers of *TACR1* rs3755468 have a higher risk of nausea after orthognathic surgery without responding sufficiently to ondansetron. For CC carriers of *TACR1* rs3755468, administering an antiemetic drug other than ondansetron may be recommended.

In 0–24 h a.a.e., CC carriers of *TACR1* rs3755468 showed a trend toward an association with metoclopramide administration (Table 4), implying that they tend to experience nausea. For mean NRS scores in 2–24 h a.a.e., CC carriers of *TACR1* rs3755468 had lower mean NRS scores than AA carriers of *CHRM3* rs2165870 (mean NRS score: CC carriers of *TACR1* rs3755468, 1.81; AA carriers of *CHRM3* rs2165870, 4.25; *P* = 3.73 × 10^− 2^; Tables 3 and 5). These results suggest that CC carriers of *TACR1* rs3755468 had lower-grade nausea. CC carriers of *TACR1* rs3755468 often have NRS scores ≤ 3, which is not fit for metoclopramide treatment even if they have nausea. This would be a reason why no significant association was found between *TACR1* rs3755468 and metoclopramide administration.

The antisense noncoding RNA *LOC105374811* gene that spans the transcription start site is expressed from around *TACR1* rs3755468 (Fig. 5 and Table S13 in Additional file 4) [31]. According to the public database, more antisense RNA *LOC105374811* is transcribed when more *TACR1* transcription is inhibited (Fig. 6) [27]. In individuals with the CC genotype of *TACR1* rs3755468, who are more prone to nausea, antisense RNA *LOC105374811* expression was lower than in the other genotypes, leading to higher *TACR1* mRNA transcription than in the other genotypes. Thus, *TACR1* rs3755468 would manipulate *TACR1* mRNA transcription through antisense RNA *LOC105374811* expression, affecting nausea.

Allele frequencies of *TACR1* rs3755468 in the present study were the following: C-allele frequency = 55%, T-allele frequency = 45%. The allele frequencies in the present study are similar to the East Asian population (C-allele frequency = 51%, T-allele frequency = 49%). Other regional populations have some variation (e.g., American population: C allele frequency = 57%, A allele frequency = 43%; European population: C allele frequency = 54%, T allele frequency = 46%, African population: C allele frequency = 64%, T allele frequency = 36%) [34]. The lower response to ondansetron and nausea risk in individuals with the CC genotype of *TACR1* rs3755468 should be examined in populations of other countries in future studies.

Hayase et al. reported that *TACR1* rs3755468 is located in the predicted estrogen response element and is associated with female sex in severe PONV [17]. Estradiol increased *TACR1* mRNA levels, which were related to an increase in ligand binding to TACR1 [37]. In the present study, *TACR1* rs3755468 showed a significant association with mean NRS scores in 0–2 h a.a.e. in the dominant model in a group of females (*P* = 3.14 × 10^− 3^), whereas no significant association was found in males (*P* > 0.05). The present study confirmed that *TACR1* rs3755468 is associated with female sex in PONV.

*Nausea mechanism related to CHRM3 rs2165870 and TACR1 rs3755468*.

In AA carriers of *CHRM3* rs2165870, the present data showed an inverse correlation between the presence of ondansetron and mean NRS scores (Table 3). In contrast, CC carriers of *TACR1* rs3755468 had a significantly higher mean NRS score in both 0–2 and 2–24 h a.a.e., regardless of the presence or absence of ondansetron (Table 5). This indicates that AA carriers of *CHRM3* rs2165870 exhibited a preventive effect of ondansetron against nausea, whereas CC carriers of *TACR1* rs3755468 exhibited only a limited preventive effect of ondansetron against nausea. Thus, these SNPs may affect nausea in a qualitatively different manner through a different mechanism or pathway.

Only around 15% of ondansetron penetrates the BBB [9]. Thus, it mainly exerts its effects outside the BBB, such as in the area postrema and vagus nerve of the upper gastrointestinal tract. In the upper gastrointestinal tract, CHRM3 is expressed at low to medium levels, whereas TACR1 has low expression at most [38]. CHRM3, whose-related nausea responded to ondansetron, is expressed at higher levels in the upper gastrointestinal tract compared to TACR1, whose-related nausea did not respond to ondansetron, consistent with the access of ondansetron on CHRM3 in the upper gastrointestinal tract outside the BBB. Ruff et al. identified a specific population of cortical γ-aminobutyric acid (GABA)ergic neurons with neuronal nitric oxide synthase and TACR1 in mice and humans, and TACR1 neurons are strong mediators of vasodilation, which is one cause of nausea [7], through feedforward excitatory pathways in the CNS [39]. Thus, TACR1 neurons in the CNS could play a central role in TACR1-related nausea. Because ondansetron hardly passes through the BBB and TACR1 is expressed at lower levels outside the CNS, ondansetron would be insufficient to suppress TACR1-related nausea. Thus, ondansetron may suppress CHRM3-related nausea predominantly outside the BBB. Altogether, our results indicate that CHRM3 and TACR1 are related to nausea in qualitatively different ways.

In conclusion, our findings suggest that AA carriers of *CHRM3* rs2165870 and CC carriers of *TACR1* rs3755468 are significantly associated with greater nausea after orthognathic surgery. The HTR3A antagonist ondansetron prevented nausea in AA carriers of *CHRM3* rs2165870 but did not in CC carriers of *TACR1* rs3755468. The present study elucidates the mechanisms of PONV and may contribute to providing tailor-made preventive care for PONV depending on high-risk genotypes of *CHRM3* rs2165870 and *TACR1* rs3755468.

The present study has a few limitations. First, the AA genotype of *CHRM3* rs2165870 was a minority among the patient subjects, with only four patients. Thus, further studies with larger sample sizes are needed. Second, this study was an observational study and thus did not directly demonstrate a causal relationship between genotype and antiemetic effect.

## Electronic supplementary material

Below is the link to the electronic supplementary material.

Supplementary Material 1

Supplementary Material 2

Supplementary Material 3

Supplementary Material 4

Supplementary Material 5

## Data Availability

All data generated or analyzed during this study are included in the published article and its supplementary information files except for those relating to nonessential personal clinical and genetic information. The undisclosed datasets used and/or analyzed during the study are available from the corresponding author upon reasonable request.

## Abbreviations

PONV: postoperative nausea and vomiting
CHRM3: cholinergic receptor muscarinic 3
TACR1: tachykinin receptor 1
SNPs: single-nucleotide polymorphisms
NRS: numeric rating scale
NTS: nucleus tractus solitarius
CNS: central nervous system
CTZ: chemoreceptor trigger zone
BBB: blood-brain barrier
ACC: anterior cingulate cortex
HTR3A: 5-hydroxytryptamine receptor 3 A
DRD2: dopamine receptor D2
OPRM1: µ-opioid receptor
ASA-PS: American Society of Anesthesiologists Physical Status
PCA: patient controlled analgesia
a.a.e.: after the anesthesia endpoint
eQTLs: expression quantitative trait loci
sQTLs: expression splicing quantitative trait loci
NCBI: National Center for Biotechnology Information
Kb: kilobases
