# Supplementary material for: Differential effects of antiemetic serotonin receptor antagonist Ondansetron on nausea associated with CHRM3 rs2165870 and TACR1 rs3755468 single-nucleotide polymorphisms
Source: Mol Brain. 2025 Jul 21;18:64. doi: 10.1186/s13041-025-01237-3 (PMC12278487; doi:10.1186/s13041-025-01237-3)
Supplement: Supplementary file 1 — Supplementary Material 1 [file 13041_2025_1237_MOESM1_ESM.docx]

**Table S1**. Effects of genetic models of *CHRM3* rs2165870 SNP on PONV risk factors.

|  | **GG + GA (n = 117)** | **AA (n = 4)** | ***P*** |
| --- | --- | --- | --- |
| Smoking history (absence / presence) | 97 / 20 | 4 / 0 | 7.31 × 10^-1^ |
| Motion sickness (absence / presence) | 76 / 41 | 2 / 2 | 1.00 |
| History of PONV (absence / presence) | 106 / 11 | 4 / 0 | 1.00 |
| Operation time (min) | 248.47 ± 62.87 | 247.50 ± 45.94 | 1.00 |
| Anesthesia time (min) | 305.72 ± 64.86 | 302.25 ± 43.45 | 1.00 |
| Total infusion volume (ml) | 1692.75 ± 374.36 | 1684.50 ± 295.46 | 1.00 |
| Blood loss during surgery (ml) | 211.01 ± 135.76 | 188.00 ± 76.67 | 1.00 |
| Total remifentanil dose (μg/kg)^a^ | 76.87 ± 22.04 | 80.01 ± 18.75 | 8.85 × 10^-1^ |
| Total intraoperative fentanyl dose (μg/kg)^a^ | 3.91 ± 0.54 | 4.03 ± 0.11 | 8.64 × 10^-1^ |
| 2 hour later postoperative fentanyl dose (μg/kg)^a^ | 1.33 ± 1.08 | 2.09 ± 1.56 | 8.10 × 10^-1^ |
| 24 hour later postoperative fentanyl dose (μg/kg)^a^ | 3.97 ± 3.31 | 5.64 ± 5.48 | 1.00 |

^a^Doses of opioids were normalized to body weight.

The data are expressed as the Mean ± SD.

**Table S2.** Effects of genetic models of *TACR1* rs3755468 SNP on PONV risk factors.

|  | **CC (n = 36)** | **CT + TT (n = 84)** | ***P*** |
| --- | --- | --- | --- |
| Smoking history (absence / presence) | 35 / 1 | 19 / 65 | 1.51 × 10^-2*^ |
| Motion sickness (absence / presence) | 19 / 17 | 58 / 26 | 1.77 × 10^-1^ |
| History of PONV (absence / presence) | 33 / 3 | 76 / 8 | 1.00 |
| Operation time (min) | 249.41 ± 48.62 | 248.12 ± 67.76 | 1.00 |
| Anesthesia time (min) | 308.61 ± 50.40 | 304.29 ± 69.68 | 8.39 × 10^-1^ |
| Total infusion volume (ml) | 1733.81 ± 344.57 | 1676.46 ± 383.74 | 8.11 × 10^-1^ |
| Blood loss during surgery (ml) | 222.47 ± 159.45 | 205.12 ± 122.42 | 1.00 |
| Total remifentanil dose (μg/kg)^a^ | 76.44 ± 17.63 | 77.07 ± 23.64 | 1.00 |
| Total intraoperative fentanyl dose (μg/kg)^a^ | 3.82 ± 0.50 | 3.95 ± 0.54 | 1.61 × 10^-1^ |
| 2 hour later postoperative fentanyl dose (μg/kg)^a^ | 1.47 ± 1.18 | 1.30 ± 1.07 | 1.10 × 10^-1^ |
| 24 hour later postoperative fentanyl dose (μg/kg)^a^ | 3.97 ± 3.04 | 4.02 ± 3.58 | 1.33 × 10^-1^ |

^*^*P* < 0.05

^a^Doses of opioids were normalized to body weight.

The data are expressed as the Mean ± SD.

**Table S3.** Association between genotypes of *CHRM3* rs2165870 SNP and metoclopramide administration (genotypic model, one-way ANOVA).

| **Hours after anesthesia endpoint** | **Metoclopramide administration** |  | **Genotypes** |  | ***P*** |
| --- | --- | --- | --- | --- | --- |
|  |  | **GG (n = 76)** | **GA (n = 41)** | **AA (n = 4)** |  |
| 0–2 | with | 1 (1%) | 0 (0%) | 0 (0%) | 1.00 |
|  | without | 75 (99%) | 41 (100%) | 4 (100%) |  |
| 2–24 | with | 15 (20%) | 10 (24%) | 3 (75%) | 7.45 × 10^-2†^ |
|  | without | 61 (80%) | 31 (76%) | 1 (25%) |  |
| 0–24 | with | 16 (21%) | 10 (24%) | 3 (75%) | 9.59 × 10^-2†^ |
|  | without | 60 (79%) | 31 (76%) | 1 (25%) |  |

^†^0.05 < *P* < 0.10

**Table S4**. Association between genotypes of *CHRM3* rs2165870 SNP and metoclopramide administration (dominant model, *t*-test).

| **Hours after anesthesia endpoint** | **Metoclopramide administration** | **Genotypes** | | ***P*** |
| --- | --- | --- | --- | --- |
|  |  | **GG (n = 76)** | **GA + AA (n = 45)** |  |
| 0–2 | with | 1 (1%) | 0 (0%) | 8.79 × 10^-1^ |
|  | without | 75 (99%) | 45 (100%) |  |
| 2–24 | with | 15 (20%) | 13 (45%) | 4.97 × 10^-1^ |
|  | without | 61 (80%) | 32 (35%) |  |
| 0–24 | with | 16 (21%) | 13 (45%) | 6.58 × 10^-1^ |
|  | without | 60 (79%) | 32 (35%) |  |

**Table S5**. Association between genotypes of *CHRM3* rs2165870 SNP and NRS scores (genotypic model, one-way ANOVA).

| **Hours after  anesthesia endpoint** | **NRS scores (mean ± SD)** | | | ***P*** |
| --- | --- | --- | --- | --- |
|  | **GG** | **GA** | **AA** |  |
| 0–2^a^ | 0.52 ± 1.51 | 0.68 ± 1.44 | 0.00 ± 0.00 | 1.00 |
|  |  |  |  |  |
| 2–24^a^ | 1.20 ± 2.25 | 1.39 ± 2.65 | 4.25 ± 2.59 | 1.08 × 10^-1^ |
|  |  |  |  |  |
| 0–24^b^ | 1.44 ± 2.45 | 1.68 ± 2.72 | 4.25 ± 2.59 | 2.18 × 10^-1^ |
|  |  |  |  |  |

^a^NRS score assessed during indicated hours after anesthesia endpoint.

^b^Maximum NRS score in 0-24 h.

**Table S6**. Association between genotypes of *CHRM3* rs2165870 SNP and NRS scores (dominant model, *t*-test).

| **Hours after  anesthesia endpoint** | **NRS scores (mean ± SD)** | | ***P*** |
| --- | --- | --- | --- |
|  | **GG** | **GA + AA** |  |
| 0–2^a^ | 0.52 ± 1.51 | 0.62 ± 1.39 | 9.88 × 10^-1^ |
|  |  |  |  |
| 2–24^a^ | 1.97 ± 2.25 | 1.64 ± 2.77 | 6.77 × 10^-1^ |
|  |  |  |  |
| 0–24^b^ | 1.44 ± 2.45 | 1.91 ± 2.80 | 6.93 × 10^-1^ |
|  |  |  |  |

^a^NRS score assessed during indicated hours after anesthesia endpoint.

^b^Maximum NRS score in 0–24 h.

**Table S7.** Association between genotypes of *TACR1* rs3755468 SNP and metoclopramide administration (genotypic model, one-way ANOVA).

| **Hours after anesthesia endpoint** | **Metoclopramide administration** |  | **Genotypes** |  | ***P*** |
| --- | --- | --- | --- | --- | --- |
|  |  | **CC (n = 36)** | **CT (n = 59)** | **TT (n = 25)** |  |
| 0–2 | with | 1 (3%) | 0 (0%) | 0 (0%) | 6.17 × 10^-1^ |
|  | without | 35 (97%) | 59 (100%) | 25 (100%) |  |
| 2–24 | with | 12 (33%) | 11 (19%) | 4 (16%) | 3.42 × 10^-1^ |
|  | without | 24 (67%) | 48 (81%) | 21 (84%) |  |
| 0–24 | with | 13 (36%) | 11 (19%) | 4 (16%) | 1.85 × 10^-1^ |
|  | without | 23 (64%) | 48 (81%) | 21 (84%) |  |

**Table S8.** Association between genotypes of *TACR1* rs3755468 SNP and metoclopramide administration (recessive model, *t*-test).

| **Hours after anesthesia endpoint** | **Metoclopramide administration** | **Genotypes** | | ***P*** |
| --- | --- | --- | --- | --- |
|  |  | **CC + CT (n = 95)** | **TT (n = 25)** |  |
| 0–2 | with | 1 (1%) | 0 (0%) | 1.00 |
|  | without | 94 (99%) | 25 (100%) |  |
| 2–24 | with | 23 (24%) | 4 (16%) | 7.63 × 10^-1^ |
|  | without | 72 (76%) | 21 (84%) |  |
| 0-24 | with | 24 (25%) | 4 (16%) | 6.60 × 10^-1^ |
|  | without | 71 (75%) | 21 (84%) |  |

**Table S9**. Association between genotypes of *TACR1* rs3755468 SNP and NRS scores (genotypic model, one-way ANOVA).

| **Hours after  anesthesia endpoint** | **NRS scores (mean ± SD)** | | | ***P*** |
| --- | --- | --- | --- | --- |
|  | **CC** | **CT** | **TT** |  |
| 0–2^a^ | 1.14 ± 2.16 | 0.29 ± 0.94 | 0.40 ± 0.94 | 3.74 × 10^-2*^ |
|  |  |  |  |  |
| 2–24^a^ | 1.81 ± 2.55 | 1.08 ± 2.53 | 1.25 ± 1.96 | 7.77 × 10^-1^ |
|  |  |  |  |  |
| 0–24^b^ | 2.33 ± 2.87 | 1.33 ± 2.44 | 1.24 ± 1.99 | 2.46 × 10^-1^ |
|  |  |  |  |  |

^*^*P* < 0.05

^a^NRS score assessed during indicated hours after anesthesia endpoint.

^b^Maximum NRS score in 0–24 h.

**Table S10.** Association between genotypes of *TACR1* rs3755468 SNP and NRS scores (recessive model, *t*-test).

| **Hours after  anesthesia endpoint** | **NRS values (mean ± SD)** | | ***P*** |
| --- | --- | --- | --- |
|  | **CC + CT** | **TT** |  |
| 0–2^a^ | 0.84 ± 1.80 | 0.40 ± 0.94 | 1.00 |
|  |  |  |  |
| 2–24^a^ | 0.92 ± 2.68 | 1.25 ± 1.96 | 1.00 |
|  |  |  |  |
| 0–24^b^ | 1.39 ± 2.36 | 1.24 ± 1.99 | 8.99 × 10^-1^ |
|  |  |  |  |

^a^NRS score assessed during indicated hours after anesthesia endpoint.

^b^Maximum NRS score in 0–24 h.
